# A Fungal P450 (CYP5136A3) Capable of Oxidizing Polycyclic Aromatic Hydrocarbons and Endocrine Disrupting Alkylphenols: Role of Trp^129^ and Leu^324^

**DOI:** 10.1371/journal.pone.0028286

**Published:** 2011-12-02

**Authors:** Khajamohiddin Syed, Aleksey Porollo, Ying Wai Lam, Jagjit S. Yadav

**Affiliations:** Department of Environmental Health, University of Cincinnati College of Medicine, Cincinnati, Ohio, United States of America; Seoul National University, Republic of Korea

## Abstract

The model white rot fungus *Phanerochaete chrysosporium*, which is known for its versatile pollutant-biodegradation ability, possesses an extraordinarily large repertoire of P450 monooxygenases in its genome. However, the majority of these P450s have hitherto unknown function. Our initial studies using a genome-wide gene induction strategy revealed multiple P450s responsive to individual classes of xenobiotics. Here we report functional characterization of a cytochrome P450 monooxygenase, CYP5136A3 that showed common responsiveness and catalytic versatility towards endocrine-disrupting alkylphenols (APs) and mutagenic/carcinogenic polycyclic aromatic hydrocarbons (PAHs). Using recombinant CYP5136A3, we demonstrated its oxidation activity towards APs with varying alkyl side-chain length (C3-C9), in addition to PAHs (3–4 ring size). AP oxidation involves hydroxylation at the terminal carbon of the alkyl side-chain (*ω*-oxidation). Structure-activity analysis based on a 3D model indicated a potential role of Trp^129^ and Leu^324^ in the oxidation mechanism of CYP5136A3. Replacing Trp^129^ with Leu (W129L) and Phe (W129F) significantly diminished oxidation of both PAHs and APs. The W129L mutation caused greater reduction in phenanthrene oxidation (80%) as compared to W129F which caused greater reduction in pyrene oxidation (88%). Almost complete loss of oxidation of C3-C8 APs (83–90%) was observed for the W129L mutation as compared to W129F (28–41%). However, the two mutations showed a comparable loss (60–67%) in C9-AP oxidation. Replacement of Leu^324^ with Gly (L324G) caused 42% and 54% decrease in oxidation activity towards phenanthrene and pyrene, respectively. This mutation also caused loss of activity towards C3-C8 APs (20–58%), and complete loss of activity toward nonylphenol (C9-AP). Collectively, the results suggest that Trp^129^ and Leu^324^ are critical in substrate recognition and/or regio-selective oxidation of PAHs and APs. To our knowledge, this is the first report on an AP-oxidizing P450 from fungi and on structure-activity relationship of a eukaryotic P450 for fused-ring PAHs (phenanthrene and pyrene) and AP substrates.

## Introduction

Polycyclic aromatic hydrocarbons (PAHs) and alkylphenols (APs) are two important groups of environmentally persistent and highly toxic chemicals that have received much attention for their slow biodegradation in the environment. Because of their persistence, these chemicals have the potential to bioaccumulate in the food-chain, causing adverse health effects in humans. PAHs are mutagenic and/or carcinogenic, and APs are endocrine-disrupting chemicals (EDCs) which can cause reproductive and other adverse health effects. Among the few microorganisms capable of oxidizing these persistent chemicals, fungi possess an extraordinary ability to completely degrade and/or mineralize (to CO_2_) these pollutants. However, little is known about the specific enzymes capable of oxidizing these chemicals as part of the biodegradation pathways in these microorganisms. Fungi have been shown to possess the capability to oxidize PAHs *via* P450-mediated mechanisms [1]–[3]. On the other hand, oxidation studies on the model AP nonylphenol (NP) in fungi [4]–[9] have implicated involvement of either extracellular oxidases [6]–[8] or hitherto unidentified intracellular enzymes [8], [9]. However, specific P450 genes/enzymes capable of oxidizing NP, or other APs, have not yet been identified from this important group of pollutant-oxidizing environmental organisms.

*Phanerochaete chrysosporium* (henceforth abbreviated as Pc), which belongs to the white rot group of the wood decaying fungi, is the most intensively studied white-rot fungus for its extraordinary ability to oxidize and/or mineralize a broad range of environmental toxicants, including PAHs and alkyl-substituted aromatics [10]–[12]. The whole genome sequencing of *P. chrysosporium* [13] had uncovered large P450 diversity, namely about 149 P450 genes [14]. Recently, using a genome-to-function strategy based on genome-wide expression profiling combined with catalytic screening of the expressed proteins, our laboratory first identified subsets of P450 genes in this fungus that are inducible by the two groups of environmentally persistent organopollutants of interest viz. APs [12] and PAHs [15]. One of the inducible fungal P450s, CYP5136A3, showed common responsiveness to both APs and PAHs, implying its catalytic versatility.

In light of these findings, the current study focuses on CYP5136A3 to assess its catalytic versatility and structure-activity relationship in terms of critical catalytic amino acid residues for biodegradation of PAHs and APs. The resulting information could improve strategies using P450-based biocatalysts for biotechnological and bioremediation applications. Additionally, considering the overall low sequence homology (12–23%) of this fungal P450 to its orthologs in humans and animals [16]–[20], it provides a unique experimental model to expand our knowledgebase on critical catalytic amino acid residues for oxidation of these xenobiotics by the eukaryotic P450s, a topic of significant current interest [21]–[23]. Indirectly, such basic information from a lower eukaryotic P450 may also provide insights into our understanding of the role of genetic polymorphisms at the active site in modulating functional diversity in the xenobiotic- and drug-metabolizing human or animal P450 enzymes.

Given the difficulties in heterologous expression of P450 genes of this fungus [24], [25], we have recently developed a yeast co-expression system, wherein *P. chrysosporium* P450s (Pc-P450s) are heterologously expressed in the active form along with the homologous cytochrome P450 reductase (Pc-CPR) in *Pichia pastoris* [15], [26]. Whole cell-based P450 assays have an advantage over *in vitro* enzyme reconstitution assays where uncoupling is the major factor that interferes in estimation of the turnover of the substrates. Hence, in this study, the whole cell biocatalyst strategy was employed using the recombinant yeast to characterize the catalytic properties of CYP5136A3 and its mutants (W129{A,L,F} and L324G) towards APs and PAHs. Phenanthrene and pyrene were used as the model compounds to understand the PAH catalysis, whereas APs with varying alkyl-chain length (C3-C9) were tested individually as model alkylphenols, in addition to the technical grade nonylphenol (tNP) that is comprised of more than 100 NP isomers containing a quaternary α-carbon [27].

We observed broad substrate specificity of CYP5136A3 towards linear APs (C3-C9) and the *t*NP mixture. The results provide experimental evidence on the role of Trp^129^ and Leu^324^ in governing the oxidation capacity and/or regio-selectivity of CYP5136A3 for oxidation of PAHs and APs.

## Materials and Methods

### Strains, media, chemicals, and growth conditions

The microbial strains, plasmids and primers used in this study are listed in Table 1. Media and growth conditions used for culturing *E. coli* TOP10 and *Pichia pastoris* KM71H (henceforth abbreviated as *PP*) were as described in the Pichia expression manual (Invitrogen, USA). PAH compounds (phenanthrene and pyrene), technical grade nonylphenol (tNP), 4*-n-*nonylphenol (4*-n-*NP) and 4*-n-*octylphenol (4*-n-*OP), 9-phenanthrol, and 1-hydroxypyrene were obtained from Sigma-Aldrich (St. Louis, MO). 4*-n-*Heptylphenol (4*-n-*HTP), 4*-n-*pentylphenol (4*-n-*PTP), 4*-n-*butylphenol (4*-n-*BP), and 4*-n-*propylphenol (4*-n-*PP) were purchased from Fisher Scientific (Pittsburg, PA). 3-Phenanthrol was purchased from Toronto Research Chemicals, Canada and 4-phenanthrol from Chiron AS, Norway.

**Table 1:** Strains, plasmids and primers used in this study.

Strain name	Genotype/Phenotype	Reference or source
*E. coli* TOP 10	F– *mcr*A Δ(*mrr*-*hsd*RMS-*mcr*BC) Φ80*lac*ZΔM15 Δ*lac*X74 *rec*A1 *ara*D139 Δ(*ara leu*) 7697 *gal*U *gal*K *rps*L (StrR) *end*A1 *nup*G	Invitrogen
*Pichia pastoris* KM71H (*PP*)	Mut^s^, Arg+	Invitrogen
*PP* C	*Pichia pastoris* KM71H containing empty vector pPICZB	15
*PP* WT	*Pichia pastoris* KM71H expresses Pc-CPR and Pc-CYP5136A3	15
*PP* W129A	*Pichia pastoris* KM71H expresses Pc-CPR and mutated Pc-CYP5136A3 (W129A)	This work
*PP* W129L	*Pichia pastoris* KM71H expresses Pc-CPR and mutated Pc-CYP5136A3 (W129L)	This work
*PP* W129F	*Pichia pastoris* KM71H expresses Pc-CPR and mutated Pc-CYP5136A3 (W129F)	This work
*PP* L324H	*Pichia pastoris* KM71H expresses Pc-CPR and mutated Pc-CYP5136A3 (L324G)	This work
**Plasmids**		
*Pc-CPR-CYP5136A3*	Binary vector construct that contains homologous reductase (*Pc-CPR*) and *CYP5136A3* in pPICZB vector background	15
*Pc-CPR-CYP5136A3* (W129A)	Binary construct that contains mutated CYP5136A3 (W129A)	This work
*Pc-CPR-CYP5136A3* (W129L)	Binary vector construct that contains mutated CYP5136A3 (W129L)	This work
*Pc- CPR-CYP5136A3* (W129F)	Binary vector construct that contains mutated CYP5136A3 (W129F)	This work
*Pc-CPR-CYP5136A3* (L324G)	Binary vector construct that contains mutated CYP5136A3 (L324G)	This work
**Primers**	**Sequence (5′ to 3′)**	
W129A	CACCGGCAAGAGTATTCTG**GCG**GCACCTGATGGCAACACG	This work
W129L	CACCGGCAAGAGTATTCTG**GTG**GCACCTGATGGCAACACGC	This work
W129F	CACCGGCAAGAGTATTCTG**TTC**GCACCTGATGGCAACACGC	This work
L324G	GTCTGCGCTCACC**GGT**GCCGGCCACGAGACGACC	This work
P1 FP	GGCCCAAAACTGACACTTTAAACGCTGTCTTGG	This work
P1 RP	GGTCTAGACTACGACCAGACATCCAACAATAGGCG	This work
P2 FP	GGTACCTTCGAAACGATGGCTGTGATCGACTATACGCTCCACGCG	This work
P2 RP & P3RP	GCGGCCGCGTAGACGGTGACGTGAAG	This work
P3 FP	GAATTCGAAACGATGGCCGTCTCTTCGTCTTCGGACG	This work

### Heterologous co-expression of CYP5136A3 and Pc-CPR in *Pichia pastoris*

A binary vector containing CYP5136A3 and homologous reductase partner (Pc-CPR) cDNAs, generated in our recent study [15] was used for heterologous expression of this P450 in *P. pastoris*.

### Site-directed mutagenesis

The binary vector containing the original coding sequence was used as a template for creating the W129A, W129L, W129F, and L324G mutants of CYP5136A3. The corresponding primers shown in Table 1 were used to create the four mutations in CYP5136A3 by using Strategene's QuikChange Lightning Multi Site-directed Mutagenesis Kit (Cat. No. 210515) following the manufacturer's instructions. Recombinant constructs (Table 1) were sequenced to select a construct showing the desired mutation for further studies.

### Transformation and selection of recombinant *P. pastoris* CYP5136A3 mutant clones

*Pme*I-linearized binary vector constructs (Table 1) containing the desired mutation in CYP5136A3 (W129A, W129L, W129F, or L324G) were transformed into *PP* using the Pichia Transformation Kit (Invitrogen). Co-presence of CYP5136A3 (mutated) and Pc-CPR in zeocin-resistant colonies resulting from the integrative transformation was confirmed by genomic DNA PCR. Site-specific genomic integration of the mutated CYP5136A3 and Pc-CPR was analyzed using three sets of primers (Table 1). The binding sites of the primers on respective DNA sequences are shown in Figure S1. Primers specific for 5′AOX region (P1FP) and for Pc-CPR (P1RP) were used to confirm the exact location of integration and the presence of Pc-CPR. Presence of CYP5136A3 was confirmed using gene-specific primers (P2FP & P2RP). Primers specific for Pc-CPR (P3FP) and CYP5136A3 (P3RP) also enabled confirmation of the intended tandem arrangement of these genes. The clones containing the mutated CYP5136A3 forms W129A, W129L, W129F, and L324G were named as *PP* W129A, *PP* W129L, *PP* W129F, and *PP* L325G, respectively. For each mutant, two positive colonies were compared for use in expression analysis; as both the colonies showed the same level of expression, data are presented for only one colony. A *PP* clone expressing the wild type (WT) form of CYP5136A3 protein along with the homologous reductase Pc-CPR (named as *PP* WT) and a *PP* clone containing the empty vector (named as *PP* C), both generated in our recent study [15], were used as positive and negative controls, respectively.

### Co-expression of CYP5136A3 enzyme and its mutant forms with the homologous cytochrome P450 reductase (Pc-CPR) in *P. pastoris*

Recombinant protein expression in the *PP* clones generated above was carried out per manufacturer's instructions (Pichia expression manual, Invitrogen) using slight modifications [15]. Briefly, a single colony from each *PP* clone was inoculated into buffered minimal glycerol (BMG) medium (100 ml) and the cultures incubated at 30°C using 250 rpm speed until the optical density (OD) at 600 nm reached 2.0. Cells were pelleted by centrifugation (5000 *g* for 5 min), washed once with buffered minimal solution and resuspended in buffered minimal methanol (BMY) medium (21 ml) containing 0.5% methanol and aminolevulenic acid (2 mM) followed by incubation at 30°C and 250 rpm for 48 h. The cells were again pelleted and resuspended in 5 ml of lysis buffer (50 mM potassium phosphate buffer pH 7.4, 20% glycerol, 1 mM PMSF, and protease inhibitor cocktail). The cells were disrupted by vortexing (15 cycles of alternate pulsing and cooling for 30 s each on ice) using an equal volume of acid-washed glass beads. The total volume was made up to 30 ml with lysis buffer and centrifuged at 5,000 *g* for 10 min at 4°C to remove cell debris. Supernatant was collected and centrifuged at 20,000 *g* for 30 min at 4°C to remove mitochondria. The resulting supernatant was again centrifuged at 100,000 *g* for 3 h to pellet. The pellet was resuspended in 4 ml of lysis buffer and the resulting microsomal preparation was used for confirming the expression of CPR and CYP5136A3 (mutant and wild type) proteins. P450 and CPR expression analysis in microsomes was done based on reduced CO-difference spectrum and NADPH-dependent cytochrome c reducing activity, respectively, as described elsewhere [28]. Homologous CPR (Pc-CPR) expression in the microsomes was confirmed by SDS-PAGE.

### Whole cell assays for P450 catalytic activity analysis

To assess the capability of wild type and mutant forms of the CYP5136A3 enzyme, we performed whole cell-based P450 oxidation activity towards phenanthrene, pyrene and APs. The method employed for analysis of the oxidation activity was the same as described previously for PAHs [15]. Briefly, a single colony from each recombinant clone (*PP* C, *PP* WT, *PP* W129A, *PP* W129L, *PP* W129F, and *PP* L324G) was cultured as described above until the optical density (OD) at 600 nm reached 2.0. Cells were pelleted and resuspended in buffered minimal (BM) medium (21 ml) containing aminolevulenic acid (2 mM). The cell suspension was subdivided equally (7 ml each) into three conical flasks. Each flask was spiked with the test xenobiotic compound dissolved in methanol. All compounds were tested at 20 ppm final concentration and the concentration of the vehicle (methanol), which was also the carbon source in the medium, did not exceed 0.5% (v/v). An uninoculated control for estimation of the initial level of the compound and for assessing the degree of any abiotic degradation was prepared using the same medium and run along side the test flasks. All treatments were performed in triplicate. The cultures were incubated (30°C, 250 rpm) for 48 h. At 24 h time-point, the cultures were spiked with methanol at 0.5% final concentration. At 48 h, 5 ml culture aliquots were removed aseptically and extracted (3×) with an equal volume of methylene chloride. The solvent extracts were dried on anhydrous sodium sulfate and resuspended in acetonitrile. Simultaneously, 1 ml of the final culture was centrifuged and the cell pellet dried to estimate the dry biomass weight, as described elsewhere [29].

### Chemical analysis and metabolite identification

Organic extracts, prepared for individual *PP* clones incubated with different xenobiotic substrate compounds as described above, were filtered through 0.45-µm glass fiber filters and analyzed using Prostar 210/215 HPLC system (Varian, Inc.) equipped with a C_18_ reverse-phase column (4.6 mm×250 mm) and a UV detector. Separation was achieved using a 20-min linear gradient of acetonitrile (ACN) in water (60% to 100% for phenanthrene; 70% to 100% for pyrene; 50% to 100% for *t*NP, 4*-n-*NP and 4*-n-*OP) or 25-min linear gradient of ACN in water (50% to 90% for 4*-n-*HTP; 50% to 80% for 4*-n-*PTP; 50% to 60% for 4*-n-*BP and 4*-n-*PP) at a flow rate of 2 ml/min. HPLC gradient for phenanthrene and pyrene metabolites was as follows: 50% methanol for 5 min, 50–75% methanol for 50 min, followed by a hold at 100% methanol for 2 min and 50% methanol for 3 min. PAHs and their metabolites were detected at 254 nm whereas APs and their metabolites were detected at 277 nm, and both groups of compounds were quantified based on standard curves generated using their known concentrations of parent compounds.

APs and their P450-hydroxylated metabolites were analyzed by liquid chromatography-electrospray mass spectrometry (LC-ESI/MS) using the LTQ-Orbitrap coupled with a surveyor MS Pump Plus (Thermo Finnigan) as described previously [15]. The solvent system was comprised of water and methanol, each supplemented with ammonium acetate at 10 mM final concentration. The LC gradient was as follows: 50% methanol for 5 min, a 45 min linear gradient of increasing methanol in water (50% to 90% for 4*-n-*NP and 4*-n-*OP; 50% to 90% for 4*-n-*HTP; 50% to 80% for 4*-n-*PTP; 50% to 60% for 4*-n-*BP and 4*-n-*PP), followed by a hold at 100% methanol for 6 min and 50% methanol for 4 min. A survey MS scan from *m/z* (100–500) at 30,000 resolution in the Orbitrap was paralleled by data dependent MS2, MS3, and MS4 scans at normalized collision energies of 25, 35 and 45, respectively. The results were analyzed using XCalibur (Thermo) and the experimental masses of metabolites were calculated by averaging 25 scans. The fragmentation patterns obtained from the MS^n^ scans were compared with those reported in the literature [30], [31].

### P450 homology modeling and ligand docking simulations

Two 3D homology-based models of CYP5136A3 were generated using the Phyre server [32] based on human microsomal P450 templates, CYP3A4 (PDB ID 1tqn) and CYP1A2 (PDB ID 2hi4). Docking simulations of the substrates were conducted with AutoDock 4 [33]. Analysis of ligand docking results was performed using POLYVIEW-MM [34]. Identification of critical amino acid residues involved in substrate recognition was done using multiple sequence alignment by ClustalW2 [35] and structure alignment by Dali [36]. Volumes of the active site cavities were calculated with CASTp [37] using a 1.4 Å probe.

### Statistical analysis

The substrate oxidation activities and the ratios of metabolites for different clones were analyzed for means and standard deviations and compared for statistical differences by Student *t* test using GraphPad QuickCalcs software package (GraphPad software, Inc. CA, USA).

## Results

### Oxidation of alkylphenols by CYP5136A3

**Figure 1:**
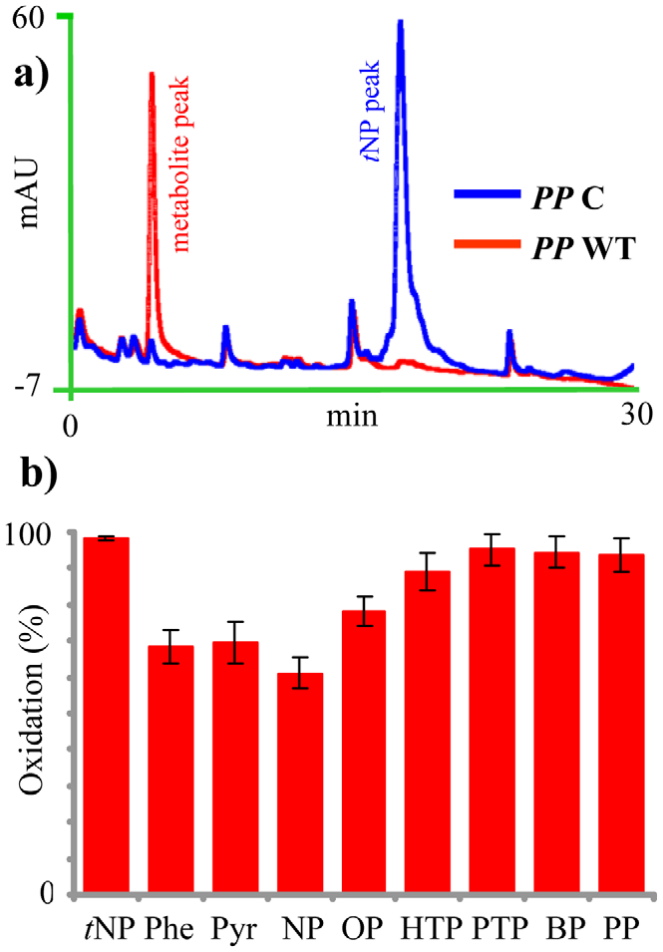
Oxidation of endocrine-disrupting alkylphenols by CYP5136A3 in whole cell catalysis using *P. pastoris* recombinant clones. **a.** HPLC analysis of the reaction extracts prepared form the cultures of *PP* C (vector only) and *PP* WT (CYP5136A3-expressing) clones incubated with technical grade nonylphenol (*t*NP). The values represent means ± standard deviations for three biological replicates. **b.** Oxidation of linear side-chain alkylphenols individually (C3-C9 chain length) and in mixture (tNP) by *PP* WT. Abbreviations: M, metabolite, NP, 4*-n-*nonylphenol; OP, 4*-n-*octylphenol; HTP, 4*-n-*heptylphenol; PTP, 4*-n-*pentylphenol; BP, 4*-n-*butylphenol; PP, 4*-n-*propylphenol. There were statistically significant (*P*≤0.05) differences between the NP oxidation rate and the oxidation rates of rest of the APs, and between the OP oxidation rate and the PTP, BP and PP oxidation rates. Differences among the oxidation rates for HTP, PTP, BP, and PP were not statistically significant.

HPLC analysis of the reaction extracts from the whole cell-based P450 assays on *t*NP showed almost complete (98%) reduction of the parent compound (*t*NP) peak and appearance of a metabolite peak in *PP* WT (*P. pastoris* clone expressing native form of CYP5136A3 along with homologous reductase partner Pc-CPR) compared to *PP* C (*P. pastoris* clone containing empty *PP*ICZB vector) (Figure 1a). There was no change in the *t*NP levels in the *PP* C and the un-inoculated control cultures, suggesting that the native *PP* P450s were not involved in *t*NP oxidation and that the observed oxidation activity in *PP* WT was due to the heterologously expressed CYP5136A3.

Analysis of the catalytic specificity of CYP5136A3 towards *t*NP was difficult as it contains a mixture of over 100 *p*-isomers [27] that generate an array of oxidized metabolites showing the overlapping separation peaks and variable hydroxylation patterns. Hence, individual linear APs (analytical grade) were employed to determine the CYP5136A3 substrate specificity and catalytic selectivity. As shown in Figure 1b, *PP* WT cells oxidized a number of APs with linear alkyl side-chain length ranging from C3 to C9, albeit to a variable extent. The C3-C5 chain-length APs including 4*-n-*propylphenol (PP), 4*-n-*butylphenol (BP), and 4*-n-*pentylphenol (PTP) were oxidized to a greater extent (93–95%) as compared to the C9 congener 4*-n-*nonylphenol (NP, 61%). APs with C8 (4*-n-*octylphenol, OP) and C7 (4*-n-*heptylphenol, HTP) alkyl-chain lengths showed 78% and 89% oxidation, respectively. The *PP* C and *PP* WT cultures grown in the presence of different APs yielded the same biomass, indicating that there is no stimulatory or inhibitory effect of heterologous P450 expression on growth rate, and that the observed oxidation activity differences are due to the expressed CYP5136A3.

### Identification of P450 oxidation products

**Figure 2:**
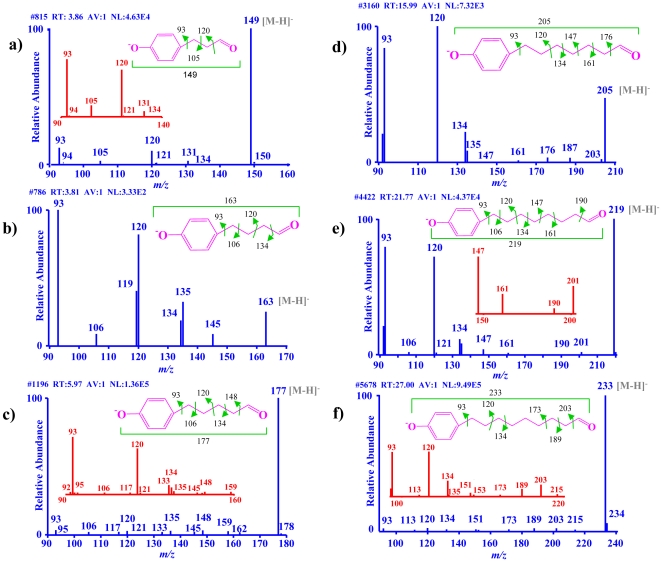
LC-ESI/MS/MS analysis of the oxidation products generated from different alkylphenols (APs). Collision-induced dissociation fragmentation pattern is shown for the metabolite formed from each of the tested APs. **a.** 4-*n*-propylphenol. **b.** 4-*n*-butylphenol. **c.** 4-*n*-pentylphenol. **d.** 4-*n*-heptylphenol. **e.** 4-*n*-octylphenol. **f.** 4-*n*-nonylphenol. Arrows in the structures of the individual AP oxidation products illustrate the observed fragmentation pattern (based on reference 31).

As observed in the HPLC chromatograms for *t*NP (Figure 1a), the LC step of the LC-ESI/MS analysis on the *PP* WT reaction extracts for the individual APs showed a single metabolite peak when compared to the control *PP* C reaction extracts (Figure S2). The identification of the metabolites was assisted by carrying out accurate mass measurement with the Oribtrap on the whole reaction extracts (Table 2). The experimentally determined *m/z* values for the precursor ion of each AP metabolite [M-H]^−^ suggested that they are aldehydes or epoxides or other structures with the same elemental composition, rather than alcohol metabolites (loss of two hydrogen atoms and gain of one oxygen atom as compared to the parent compounds) (Table 2). In order to further confirm the identity of the AP oxidation products as AP-aldehydes, MS*^n^* was employed to locate the position of the functional group in the metabolites. As demonstrated by the fragment ions of different AP metabolites under collision-induced dissociation, fragmentation happened between the aromatic moiety and the alkyl side-chain between the carbon atoms at C1-C2 (105/106), C2-C3 (120), C3-C4 (134/135), C4-C5 (147/148), C5-C6 (161), C6-C7 (173/175), C7-C8 (189/190) and C8-C9 (203) (Figure 2). In addition, the presence of fragment ions with an *m/z* value corresponding to the loss of CHO (29 amu) from the parent ion of all the AP metabolites suggested that hydroxylation occurred at the terminal carbon atom of the alkyl-chain in different AP metabolites (Figure 2). The formation of aldehyde rather than epoxide metabolites is further supported by the facts that the substrate is a saturated alkane chain with no double bond [38] and that similar terminal (*ω*-) hydroxylations in yeasts yield aldehyde metabolites due to the activity of the resident alcohol dehydrogenases on the P450-hydroxylated intermediate [39]. Altogether, the molecular mass determined by mass measurements (Table 2) and the metabolite fragmentation patterns (Figure 2) confirmed that the aldehyde group is present at the terminal carbon of the alkyl-chain in the metabolites of different APs (Figure 2 and Table 2).

**Table 2:** LC-ESI/MS/MS analysis of the metabolites formed by the CYP5136A3 monooxygenation reaction on different alkylphenols (APs).

Parent compound			Metabolite							
Name	Chemical formula	No. of carbons in alkyl side-chain	No. of peaks	Rt(min)	Precursor ion m/z[M-H]^−^(AV:25)^a^	MW	Deduced chemical formula for the metabolite	MW of the deduced formula (theoretical)	LC-ESI/MS^n^ fragmentation pattern	Metabolite name
4*-n-*PP	C_9_H_12_O	3	1	3.9	149.0616	150.0689	C_9_H_10_O_2_	150.0675	Fig. 2a	3-(4-hydroxy phenyl)propanal
4*-n-*BP	C_10_H_14_O	4	1	3.5	163.0771	164.0844	C_10_H_12_O_2_	164.0831	Fig. 2b	4-(4-hydroxy phenyl)butanal
4*-n-*PTP	C_11_H_16_O	5	1	5.9	177.0930	178.1003	C_11_H_14_O_2_	178.0988	Fig. 2c	5-(4-hydroxy phenyl)pentanal
4*-n-*HTP	C_13_H_20_O	7	1	16.0	205.1241	206.1314	C_13_H_18_O_2_	206.1301	Fig. 2d	7-(4-hydroxy phenyl)heptanal
4*-n-*OP	C_14_H_22_O	8	1	22.4	219.1458	220.1531	C_14_H_20_O_2_	220.1457	Fig. 2e	8-(4-hydroxy phenyl)octanal
4*-n-*NP	C_15_H_24_O	9	1	26.9	233.1618	234.1691	C_15_H_22_O_2_	234.1614	Fige. 2f	9-(4-hydroxy phenyl)nonanal

a Accurate mass determination was carried out by averaging 25 (AV: 25) scans across the full width half maximum of the extracted ion profile. Abbreviations: 4*-n-*NP, 4*-n-*nonylphenol; 4*-n-*OP, 4*-n-*octylphenol; 4*-n-*HTP, 4*-n-*heptylphenol; 4*-n-*PTP, 4*-n-*pentylphenol; 4*-n-*BP, 4*-n-*butylphenol; 4*-n-*PP, 4*-n-*propylphenol; Rt, retention time.

### Structural analysis of CYP5136A3 protein

Three-dimensional homology-based models of CYP5136A3 were built using the Phyre web-server [32] based on appropriate P450 templates. Among the 10 top-scoring templates selected by Phyre, 2 templates corresponded to the mammalian P450s known to oxidize PAHs [40] and other aromatic xenobiotics. These were human microsomal P450s, CYP3A4 (PDB ID 1tqn) and CYP1A2 (PDB ID 2hi4), which showed 22% and 17% overall amino acid sequence identity to CYP5136A3, respectively. Of these two human P450s, 3A4 has a significantly larger active site cavity [21] compared to 1A2 [22]. Structural alignment of the CYP5136A3 models showed 3.2 Å RMSD (Figure S3). The model based on the 3A4 template was estimated to have the cavity with a volume of 5720.8 Å^3^. This volume includes the putative substrate access channel. The volume of the cavity in the second model (based on 1A2) was estimated to be 2428.2 Å^3^, as this model did not indicate presence of a substrate access channel. In this study, we have chosen the 3A4-based model, as this template had a relatively higher overall sequence identity with CYP5136A3 as compared to other templates. In addition, sequence homology within the SRS regions between CYP5136A3 and the mammalian CYP3A4 template constitutes 34% sequence identity (“identity”) and 58% conservative identity (“similarity”). Major elements of the CYP5136A3 topology are indicated in Figure S4, enabling the approximate localization of the six substrate recognition sites (SRS), SRS1-SRS6, typically present in a P450 protein [41], [42]. The model lacks the β4 sheet of the template [21], which is replaced by the unstructured loops.

**Figure 3:**
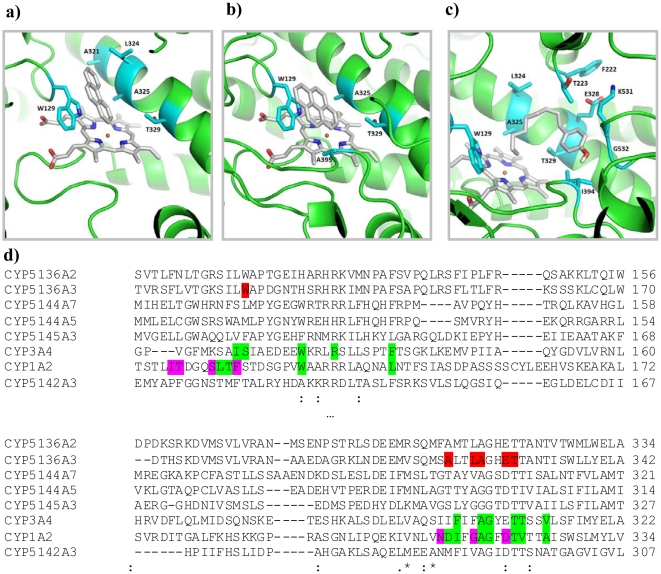
Putative binding modes of the xenobiotic substrates obtained from ligand docking simulation in the CYP5136A3 homology-based model. **a.** Phenanthrene. **b.** Pyrene. **c.** 4-*n*-Nonylphenol. Residues colored cyan are in contact with the substrate. **d.** Two fragments of the same multiple sequence alignment of P450s that are known to be PAH oxidases. Color highlighting: green– residues contacting heme, magenta– residues in contact with co-crystallized ligand, red– a union of residues in contact with the three test substrates.

The CYP5136A3 3D model was subsequently used for ligand docking simulations by AutoDock 4 [33]. Structures of representative substrates, specifically phenanthrene, pyrene, and 4*-n-*nonylphenol, were docked into the putative active site cavity. Contacting residues were identified from the top-scoring poses of the substrates (Figure 3a–c) using the POLYVIEW-MM server [34]. To pinpoint the residues critical for substrate recognition, multiple sequence alignment (MSA) was performed between the mammalian PAH oxidases (CYPs 1A2 and 3A4) and the novel PAH-oxidizing fungal P450s recently identified in our laboratory [15] (Figure 3d). Out of 7 residues found to contact all three docked substrates (W129, A321, L324, A325, E328, T329, A395; Figure 3d, residues shaded in red), 3 residues show significant variation across the aligned orthologs (W129/SRS-1, A321/SRS-4, L324/SRS-4), 3 residues are highly conserved (A325, E328, T329, all located in helix I/SRS-4), and 1 residue (A395, located in close proximity to SRS-5; not shown) has no counterpart in other enzymes according to MSA. A395 is located in the flexible loop region of the model, beyond the substrate recognition site. Based on our analysis of the resolved crystal structures of 3A4 [21] and 1A2 [22], we reasoned that the three highly conserved residues, being also involved in heme binding, may play a dual role (as per the crystal structure analysis; Figure 3d, residues shaded in green); hence the mutation of these residues may abolish heme binding, an undesirable effect for this study. The same consideration related to A321. Therefore, we selected the remaining two residues (W129 and L324) for site-directed mutagenesis (SDM) from the list of residues that showed significant variation.

### Comparison of P450 and CPR expression levels for WT versus mutant clones of CYP5136A3

The effects of the mutations in terms of the expression levels of the P450 (CYP5136A3) and its oxidation capacity towards xenobiotic compounds were investigated by comparing the mutant *PP* clones with the control-positive (*PP* WT) and control-negative (*PP* C) clones.

**Figure 4:**
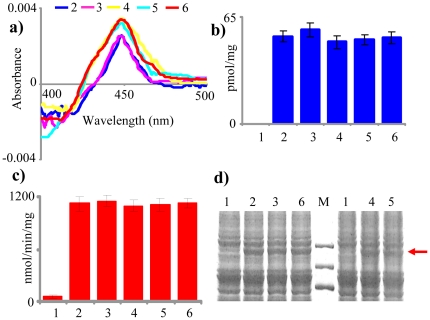
Recombinant co-expression and activity analysis of CYP5136A3 or its mutant forms (W129A, W129L, W129F, and L324G) and the homologous P450 reductase (Pc-CPR) partner in *P. pastoris*. Expression characteristics of the recombinant enzymes were assessed in the yeast microsomes. **a.** characteristic reduced CO-difference spectra (the presented spectrum was chosen from a set of eight runs for each P450; the maximum absorbance value varied from 446 nm to 451 nm with 448 nm being the most frequently recorded i.e. the mode value). **b.** P450 content. **c.** CPR activity (NADPH-dependent cytochrome c reducing activity). **d.** SDS-PAGE analysis of the microsomal protein preparations from recombinant *P. pastoris* clones. Lanes labeled 1 through 6 represent microsomal fractions prepared form *PP* C, *PP* WT, *PP* W129A, *PP* W129L, *PP* W129F, and *PP* L324G recombinant clones, respectively. CPR activity analysis was performed in triplicate; the means and standard deviations were calculated. M indicates protein molecular weight marker. Expression of Pc-CPR is shown with an arrow. The differences in P450 quantities and CPR activities in microsomes across the recombinant clones were not statistically significant (*P*≥0.05).

Microsomes from the *PP* clones expressing WT, W129A, W129L, W129F, and L324G forms of CYP5136A3 showed characteristic reduced CO-difference P450 spectrum as opposed to no such spectrum observed in the microsomes prepared from *PP* C (Figure 4a). CYP5136A3 WT and mutated forms showed the maximum absorption peak at 448 nm (Figure 4a). Comparable levels of expression of the P450 protein (in the range of 49–53 pmol of P450/mg of microsomal protein) were observed in microsomes prepared from both *PP* WT and *PP* clones containing the mutated CYP5136A3 (Figure 4b).

Microsomes from all five *PP* clones (*PP* WT, *PP* W129A, *PP* W129L, *PP* W129F, and *PP* L324G) showed high CPR activity at comparable levels (Figure 4c), in the range of 1095–1151 nmol/min/mg protein. In comparison, the *PP* C microsomes showed negligible native yeast CPR activity (50 nmol/min/mg protein). In order to further verify that the higher levels of CPR activity in the recombinant clones are due to the expression of cloned Pc-CPR, SDS-PAGE profiles of the microsomal proteins were compared. An intense band corresponding to Pc-CPR (Figure 4d) in the microsomal preparations from the *PP* clones WT, W129A, W129L, W129F, and L324G as opposed to no such band in the *PP* C microsomes confirmed that the high CPR activity in the recombinant *PP* clones (WT, W129A, W129L, W129F, and L324G) is due to actual expression of the cloned Pc-CPR.

### Comparison of the xenobiotic-oxidizing activity of WT versus mutant forms of CYP5136A3

Whole cell catalysis assays using the *PP* WT and mutant *PP* clones allowed assessment of the catalytic role of Trp^129^ and Leu^324^ residues of CYP5136A3 in the oxidation of different APs and PAHs (phenanthrene and pyrene).

**Figure 5:**
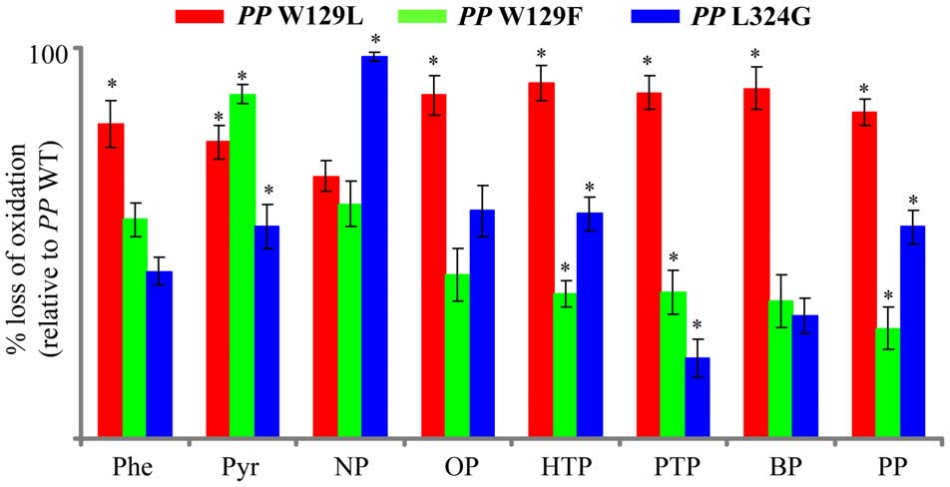
Relative PAH- and AP-oxidizing activities of the mutant forms of CYP5136A3. Percent loss of oxidation activity in the mutant clones (*PP* W129L, *PP* W129F and *PP* L324G) relative to the *PP* WT clone (which expresses wild type CYP5136A3), was calculated by considering the *PP* WT oxidation of PAHs and APs as 100%. The values represent means ± standard deviations for three biological replicates. Asterisks indicate values that are statistical significant (*P*≤0.05) for a given compound between recombinant clones. Abbreviations: Phe, phenanthrene; Pyr, pyrene; NP, 4*-n-*nonylphenol; OP, 4*-n-*Octylphenol; HTP, 4*-n-*heptylphenol; PTP, 4*-n-*pentylphenol; BP, 4*-n-*butylphenol; PP, 4*-n-*propylphenol.

HPLC profiles of the reaction extracts showed no considerable effect (p>0.05) of the W129A mutation on the extent of oxidation of either PAHs or APs (Figure S5). However, unlike W129A, W129L and W129F mutations caused significant decrease (p<0.05) in the oxidation of both PAHs and APs (Figure 5). *PP* W129L showed higher reduction (81%) in phenanthrene oxidation activity as compared to *PP* W129F (56%), and both clones showed a significant decrease (76% by *PP* W129L and 88% by *PP* W129F) in pyrene oxidation (p<0.05). For APs, *PP* W129F showed a substantial loss in APs oxidation (60% for C9 AP and 28–41% for C3-C8 APs) whereas *PP* W129L showed an almost complete loss of oxidation activity for C3-C8 APs (83–90%) and a comparable loss in oxidation activity for C9 AP.

The L324G mutant caused a nearly complete impairment of NP oxidation (95%) when compared to the WT form. Furthermore, it showed almost 50% lower level of oxidation activity in case of OP, HTP and PP as compared to WT (Figure 5). Oxidation levels for medium-chain APs, such as PTP and BP, were decreased to 21% and 36% (compared to WT form), respectively. The mutant clones (*PP* W129A, *PP* W129F, *PP* W129L, and *PP* L324G) yielded the same biomass as the wild type (*PP* WT) and the control (*PP* C) yeast clones, indicating (i) no adverse effect of the P450 mutations on the rates of growth, and (ii) that the oxidation activity differences among the clones are due to the P450 mutations.

### Regio-selectivity of WT versus mutant forms of CYP5136A3

The metabolite profiles for PAHs (phenanthrene and pyrene) and APs (varying in alkyl-chain lengths) were compared between WT and the mutants. The HPLC and LC-ESI/MS analysis of the xenobiotic reaction extracts prepared using *PP* W129A, *PP* W129L, *PP* W129F, and *PP* L324G showed differences in metabolite profiles for phenanthrene and pyrene as compared to *PP* WT (Table 3).

**Table 3:** Comparison of the metabolite profiles in *P. pastoris* clones expressing the wild type form (*PP* WT) and the mutant forms (*PP* W129A, *PP* W129L, *PP* W129F, and *PP* L324G) of CYP5136A3.

Clone	Phenanthrene metabolites^a^			3∶4 ratio^b^		Pyrene metabolites^a^	
	3-OH phe	4-OH Phe	9-OH Phe	3-OH Phe	4-OH Phe	1-OH Pyr	2-OH Pyr^c^
***PP*** ** WT**	5.8(±0.1)	40.7(±2.4)	53.5(±2.5)	12.5	87.5	36(±2.7)	64.0(±3.4)
***PP*** ** W129A**	4.4(±0.3)	32.0^*^(±1.9)	63.6^*^(±2.3)	12.1	87.9	24.7^*^(±2.5)	75.3^*^(±3.1)
***PP*** ** W129F**	6.6(±0.5)	45.4^*^(±1.1)	48.0^*^(±0.6)	12.8	87.2	-	-
***PP*** ** W129L**	3.6(±0.5)	47.0^*^(±1.9)	49.4^*^(±1.2)	6.8	93.2	26.5^*^(±1.3)	73.5^*^(±2.1)
***PP*** ** L324G**	0.1^*^(±0.02)	0.9^*^(±0.5)	99.0^*^(±0.6)	7.2	92.8	17.5^*^(±2.7)	82.5^*^(±2.3)

a For each metabolite, the observed HPLC count value was converted into metabolite concentration using the standard curve generated with the known quantity of the metabolite (X-axis) and its HPLC count value (Y-axis). The proportion of each metabolite (%) was calculated from the total amount of metabolites (observed for each oxidation reaction) which was considered as 100%. Values represent means ± standard deviations (in parenthesis) based on three biological replicates. Asterisks indicate values that are statistical significant (*P*≤0.05) compared to *PP* WT. Formation of 3-OH Phe is a random event [50], [52] and hence it is not an indication of regio-selectivity by the mutants. All clones (*PP* WT, *PP* W129A, *PP* W129F and *PP* L324G) showed comparable product recovery (89–92% product recovery) for phenanthrene. For pyrene, HPLC absorbance values were used to quantify the amounts of the oxidation products in *PP* W129A, *PP* W129L, and *PP* W324G compared to the *PP* WT sample and the product recoveries are shown in the table. Due to low levels of pyrene oxidation by *PP* W129F, the metabolite level was below detection limit. Abbreviations: 3-OH Phe, 3-phenanthrol; 4-OH Phe, 4-phenanthrol; 9-OH Phe, 9-phenanthrol; 2-OH Pyr, 2-hydroxypyrene; 1-OH Pyr, 1-hydroxypyrene.

b The ratio between 3-phenanthrol and 4-phenanthrol was calculated on a scale of 100. Due to low levels of pyrene oxidation by *PP* W129F, the metabolite level was below detection limit. The mechanism of oxidation of phenanthrene followed the NIH shift mechanism as all the mutants showed higher levels of 4-phenanthrol compared to 3-phenanthrol. See text for details.

c In light of the commercial non-availability of an authentic standard for 2-pyreneol, we used the molecular mass and the HPLC elution profile criteria to tentatively identify the second peak as 2-pyrenol [15]; both profiles matched with the CYP101 mutants' pyrene oxidation profile [72], where 1-pyrenol had longer retention time as compared to 2-pyrenol.

WT enzyme oxidized phenanthrene into three metabolites *viz*. 3-phenanthrol, 4-phenanthrol and 9-phenanthrol in a ratio of 5.8∶40.7∶53.5. A different ratio (4.4∶32.0∶63.6) was observed in case of W129A mutant indicating that this mutation caused a decrease in the formation of 3-phenanthrol (−1.4%) and 4-phenanthrol (−8.7%), and an increase in the formation of 9-phenanthrol (+10.1%). Interestingly, in contrast with W129A, the W129F mutation (which showed a 3-, 4-, 9-phenanthrols ratio of 6.6∶45.4∶48.0) caused a decrease in 9-phenanthrol (−5.5%) level, and an increase in 3-phenanthrol (+0.8%) and 4-phenanthrol (+5.5%) levels compared to the WT form. W129L also followed the same trends as W129F and caused a higher level of 4-phenanthrol formation (+6.3%) and a lower level of 9-phenathrol (+4.1%) formation as compared to WT. On the other hand, the L324G mutant showed a large difference in the ratio of phenanthrene metabolites as compared to W129A and WT; the observed 0.1∶0.9∶99.0 ratio indicated an increased formation of 9-phenanthrol (+45.5%) and a decreased formation of 3- and 4-phenanthrols (−5.7% and −39.8%, respectively) when compared to WT. Interestingly, when calculated separately, just for the 3-phenanthrol∶4-phenanthrol ratio, it was comparable among the WT, W129A, and W129F, but appeared shifted for L324G towards a higher proportion of 4-phenanthrol (Table 3).

Analysis of the pyrene reaction extracts further suggested differences in the metabolites ratio between *PP* WT and the mutants (Table 3). WT oxidized pyrene into two metabolites, 1-hydroxypyrene and 2-hydroxypyrene in a ratio of 36∶64 suggesting that the formation of 2-hydroxypyrene was favored over 1-hydroxypyrene formation. Notably, mutations at both positions (W129A, W129L, and L324G) led to a further increase in the levels of 2-hydroxypyrene formation (Table 3), resulting in the following ratios between the two metabolites: 24.7∶75.3 (W129A), 26.5∶73.5 (W129L), and 17.5∶82.5 (L324G). *PP* W129F showed no detectable metabolites in the HPLC profiles due to low levels of pyrene oxidation by this mutant.

## Discussion

### CYP5136A3 as an AP alkyl side-chain *ω*-hydroxylase

Alkylphenols have endocrine-disrupting activity, in that they are known to mimic mammalian estrogenic hormones [43]. This activity is attributed to both alkyl side chains and phenolic moieties, which highlights the importance of biodegradation of the side-chains of these estrogenic compounds. Previous metabolite identification-based studies on certain species of yeast and fungi have shown their ability to metabolize the model AP congener 4*-n-*NP via terminal oxidation of the alkyl side-chain followed by a β-oxidation pathway [4], [5], [9]. The remaining aromatic ring is then cleaved and used as a carbon source [9]. However, specific enzymes involved in the terminal oxidation of the alkyl side-chain have not been identified in these lower eukaryotes.

Our recent report on inducibility of CYP5136A3 by *t*NP in *P. chrysosporium* [12] and the data in this study on complete oxidation of *t*NP by recombinant CYP5136A3 collectively suggest that this compound is both an inducer and a substrate for this enzyme. The extent and rate of the oxidation (almost 100% oxidation of 20 ppm *t*NP in 48 h) pointed to the promising potential of the CYP5136A3 enzyme as a catalyst for use in bioremediation of *t*NP. Our results showed that CYP5136A3 possesses a broad substrate-specificity towards linear APs with alkyl-chain length ranging form C3 to C9. Based on the results of this work, CYP5136A3 appears to preferentially target primarily short alkyl-chain APs, such as PTP, BP, and PP, over the longer alkyl-chain APs, such as NP and OP. Activity towards HTP was moderate (intermediate between the short and the long alkyl-chain containing APs).

**Figure 6:**
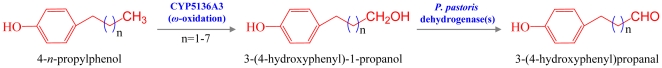
Schematic representation of the pattern of oxidation of alkylphenols by *P. pastoris* cells expressing CYP5136A3. Alkylphenols were oxidized at the terminal carbon atom (*ω*-oxidation) of their alkyl side-chain by the CYP5136A3 activity and the resulting *ω*-hydroxylated AP metabolites were further oxidized to their respective *ω*-aldehydes by the action of the known yeast host dehydrogenase activity.

In the whole cell P450 biocatalysis assays for APs, an aldehyde metabolite accumulated, rather than the expected hydroxylated AP, suggesting that the resident host yeast (*P. pastoris*) dehydrogenase activity is involved in further oxidation of the hydroxylated AP metabolite generated by the recombinant fungal P450 (Figure 6). This is analogous with the *n*-alkane ω-oxidation pathway in alkane-utilizing yeasts [39] that proceeds *via* removal of terminal carbon from the *n*-alkane chain by successive hydroxylation and oxidation reactions mediated by P450 monooxygenase, alcohol dehydrogenase, and aldehyde dehydrogenase, respectively. In order to confirm the CYP5136A3 catalytic specificity towards APs, identifying the location of the functional group (aldehyde) in AP-metabolite is critical. Mass fragmentation of the metabolite showed that the aldehyde group is located at the terminal carbon of the alkyl-chain of each tested AP metabolite, confirming that CYP5136A3 is an alkyl side-chain *ω*-hydroxylase (Figure 6). The observed *ω*-hydroxylation of varying chain-length (C3-C9) APs implied that this P450 plays a role in fungal biodegradation of APs via sequential shortening of the side-chain. This is consistent with our whole fungus-based studies on another structurally-related compound, the linear alkylbenzene sulfonate by *P. chrysosporium*, wherein sequential shortening of the 12-carbon linear alkyl chain was demonstrated [44].

### Catalytic role of Trp^129^ and Leu^324^ residues of CYP5136A3 in oxidation of APs and PAHs

**Figure 7:**
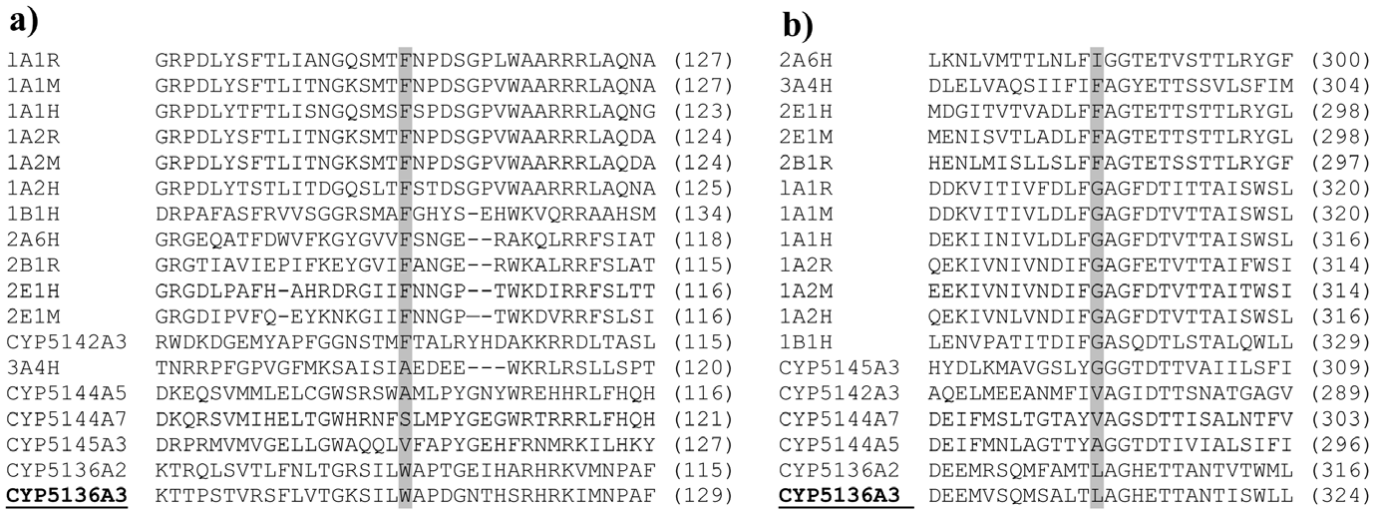
Amino acid sequence alignment of the eukaryotic P450s capable of oxidizing polycyclic aromatic hydrocarbons (PAHs). Context amino acid sequences corresponding to W129 (a) and L324 (b) of CYP5136A3 are shown (the specific amino acid position in each P450 corresponding to these residues is included in the parenthesis). Mammalian P450 sequences for human (H), rat (R), and mouse (M) were retrieved from the Nelson P450 website (http://drnelson.uthsc.edu/CytochromeP450.html). Model white-rot fungus *P. chrysosporium* P450s capable of oxidizing PAH compounds per our recent report [15] were used for comparison with the mammalian P450s. Amino acid residues corresponding to W129 and L324 of CYP5136A3 are highlighted in bold and are shaded.

A homology-based 3D model of CYP5136A3, combined with ligand docking simulations, suggested that Trp^129^ and Leu^324^ residues play a key role in the catalytic activity of this enzyme. ClustalW2 alignment of the amino acid sequence of CYP5136A3 with those of the mammalian orthologs showed limited variability in mammals at the positions corresponding to CYP5136A3 W129 and L324 (Figure 7). Specifically, at position 129, the majority of mammalian enzymes harbor Phe, whereas fungal P450s display a broad variability. Notably, human CYP3A4 (with very broad substrate specificity), used as a template for CYP5136A3 model, also differs at 129 from the other aligned human P450s, supporting our hypothesis that this is one of the critical positions responsible for versatile substrate recognition. At position 324, mammalian enzymes have either a hydrophobic and aromatic amino acid (Phe), or a polar amino acid (Gly), whereas fungal P450s have hydrophobic aliphatic amino acid residues only (Ala, Val, and Leu). In this context, homology-based modeling and site-directed mutagenesis reports on mammalian P450s have shown that these residues are critical in substrate binding and oxidation for various substrates [45]–[49], although no studies are available on alkylphenols as well as on the tested PAH compounds (phenanthrene, pyrene).

In order to assess the role of Trp^129^ and Leu^324^ in the oxidation of PAHs and APs, we replaced Trp^129^ with amino acids of increasing hydrophobic gradient (Ala, Phe, and Leu with the Kyte-Doolittle hydropathy indices 1.8, 2.8, and 3.8, respectively, as opposed to −0.9 for Trp) and hydrophobic Leu^324^ with polar Gly (a conserved residue within the selected mammalian PAH-oxidizing P450s). Collectively, these mutations were intended to confirm our homology model-based predictions on the catalytic role of these amino acids. This also allowed us to understand the catalytic relevance (specificity and/or regio-selectivity) of these residues in the fungus compared to their mammalian counterparts in this catalytic group of P450s (Figure 7).

Mutation of Trp^129^ (W129A, W129L, and W129F) and Leu^324^ (L324G) residues in CYP5136A3 caused no adverse effect on the active expression of the mutant forms of CYP5136A3 as revealed by the unaltered typical CO-difference spectrum (Figure 4). Comparable levels of redox partner (Pc-CPR) expression in the mutant clones (*PP* W129A, *PP* W129L, *PP* W129F, and *PP* L324G) *versus* the control clone (*PP* WT) enabled uniform supply of electrons for the wild type and mutant forms (W129A, W129L, W129F, and L324G) of CYP5136A3. This directly supported the interpretation that the observed differences in the oxidation profile of PAHs and APs are due to the effect of mutations and not because of the differences in supply of electrons for the oxidation reaction, nor of the unfolding of the enzyme.

### Oxidation of Alkylphenols

Although information on P450 oxidation of alkylphenols is available for certain mammalian P450s, studies on the P450 structure-activity relationship with respect to this group of compounds have not been reported. Here we identified two amino acid residues that play a key role in the oxidation of alkylphenols by the P450 enzyme CYP5136A3 from the model white-rot basidiomycetous fungus *P. chrysosporium*. Substitution of Trp^129^ with Leu (W129L) or Phe (W129F) caused loss of oxidation of APs. W129L was deleterious, as it resulted in complete loss of AP oxidation. This suggested that W129 of CYP5136A3 plays a key role in the oxidation of APs. Like W129L and W129F, the Leu^324^ mutation (L324G) was also found to be critical for the AP oxidation activity. Notably, L324G mutation caused a complete loss of oxidation activity towards 4*-n-*nonylphenol, the most environmentally recalcitrant congener among the APs, indicating a key role of L324 residue in alkylphenol oxidation activity. All these mutations had no effect on regio-selectivity of hydroxylation of APs as the metabolite profile was the same for the mutant forms and the WT form. This is the first report across the phylogenetic ranks on identification of critical residues in a P450 enzyme involved in AP oxidation.

### Oxidation of Phenanthrene

Phenanthrene, a three-ring PAH, has been used as a model compound in various studies on the mammalian metabolism of PAHs due to the presence of “K-region”, a common structural feature of carcinogenic PAHs [50], [51]. Recombinant CYP5136A3 oxidized phenanthrene into three hydroxylated metabolites 3-, 4-, and 9-phenanthrol, suggesting the formation of two *trans*-dihydrodiols, (3,4 and 9,10). The three phenanthrols detected in the study were likely formed either by dehydration of the *trans*-dihydrodiols [52] or by rearrangement of the postulated arene oxides [50]. Given that 1- and 2-phenanthrols, the products of phenanthrene oxidation at 1,2-position, were not formed by CYP5136A3, there is a major difference between this enzyme and P450s from other eukaryotes. For instance, the zygomycete fungus *Cunninghamella elegans* [53], marine animals, such as lobsters and sharks [54], and mammals, have been reported to oxidize phenanthrene at the three alternative (3,4-, 9,10-, and 1,2-) positions [55]–[60]. Furthermore, unlike mammalian P450s (human CYPs 1A1, 2A6, 2E1, 3A4, and 1B1; rat CYPs 1A1, 1A2, and 2B1; mouse CYPs 1A1, 1A2, and 2E1; guinea pig CYPs [55] and P450s from marine animals [54]), where phenanthrene *trans*-9,10-dihydrodiol is the predominant metabolite, CYP5136A3 showed almost comparable proportions of *trans*-9,10-dihydrodiol and *trans*-3,4-dihydrodiol (54∶46), as indicated by the relative amounts of 9-phenanthrol *versus* 3- and 4-phenanthrols (Table 3). Collectively, this suggests that CYP5136A3 is unique among the phenanthrene-oxidizing P450s as its forms only two *trans*-dihydrodiols, and that these are in comparable proportions unlike in other eukaryotes. Interestingly, the ratio between 3- and 4-phenanthrols followed the NIH shift mechanism [50] as the amount of 4-phenanthrol was relatively higher than that of 3-phenanthrol (Table 3).

While substitution of Trp^129^ with Ala (W129A) had no effect on the oxidation activity of CYP5136A3 towards phenanthrene, substitution with Leu (W129L) and Phe (W129F) decreased the oxidation of phenanthrene, with nearly a complete loss of oxidation for W129L. Although W129A had no effect on the overall oxidation level of phenanthrene, it altered the regio-selectivity of CYP5136A3 towards 9-phenanthrol formation. Interestingly, in contrast with the W129A mutation, W129F and W129L mutations led to high levels of 4-phenanthrol and low levels of 9-phenanthrol formation. This suggests that Trp^129^ of CYP5136A3 plays a key role in regio-selective oxidation of phenanthrene at both *K*-region (9,10- C position) and non-*K* region (3,4- C position).

L324G reduced the phenanthrene oxidation activity by half and favored the formation of 9-phenanthrol compared to the WT enzyme. While formation of 3- and 4-phenanthrols was slightly lowered in W129A, L324G mutation led to negligible levels of these phenanthrols. However, the ratio between 3- and 4-phenanthrols in the W129A, W129F, and L324G mutants still followed the NIH shift mechanism as in case of the WT form of the enzyme (Table 3). This suggested Trp^129^ and Leu^324^ play a key role in the positioning of the phenanthrene towards the CYP5136A3 heme oxidation center. L324G converts the fungal P450 regio-selectivity into the mammalian P450-like regio-selectivity for phenanthrene (as indicated by altered dominance of the 9,10- diol over the 3,4- diol form). Taken together, our observations suggest that the two residues Trp^129^ and Leu^324^ are critical in modulating the regio-selectivity of PAH oxidation.

### Oxidation of Pyrene

CYP5136A3 oxidized pyrene into two monohydroxylated metabolites, 1-pyrenol and 2-pyrenol in a ratio of 64∶36 (Table 3) suggesting this enzyme's interaction with the non-*K* region of pyrene. Interestingly, to our knowledge, no other P450 across the prokaryotes and eukaryotes has been reported to yield any additional pyrenol (other than 1-pyrenol) from pyrene [61]–[70]. Nevertheless, site-directed mutagenesis of P450cam (CYP101) from *Pseudomonas putida* [71] was shown to enable generation of both pyrenols; the CYP101 mutants also formed pyrene quinones in addition to pyrenols [71]. However, CYP5136A3 and CYP101 mutants differ in the ratio for the two pyrenols. Various CYP101 mutants formed 1- and 2- pyrenols and the pyrene quinones in a ratio of 84∶10∶6 (Y96A), 75∶1∶24 (Y96F), 35∶0.5∶64.5 (F87A-Y96F) and 37∶1∶62 (F87L-Y96F), suggesting the predominance of 1-pyrenol in the oxidation product mixtures [71]. In contrast, CYP5136A3 showed lower levels of 1-pyrenol as compared to 2-pyrenol. This suggested that CYP5136A3 is unique among the pyrene-oxidizing P450s because it forms two monohydroxylated products (pyrenols) and relatively low amount of 1-pyrenol. As observed for phenanthrene, W129A mutation had a negligible effect on the levels of pyrene oxidation, unlike the other hydrophobic amino acid substitutions at this position which caused 75% (W129L) and 88% (W129F) loss of pyrene oxidation. L324G mutation caused decrease in the pyrene oxidation levels by half. These observations collectively suggest that both W129 and L324 residues are critical in the oxidation of PAHs. Although mutations at Trp^129^ (W129A and W129L) and Leu^324^ (L324G) favored the formation of 2-pyrenol, its amount was predominant in L324G as compared to W129A and W129L, suggesting the role of these amino acid residues in the regio-selectively of pyrene (1- C position) oxidation. Because of the low level of pyrene oxidation by W129F mutation and difficulty in quantification of its pyrene metabolites, the role of this substitution in regio-selective oxidation of pyrene is not clear.

In conclusion, this study led to functional characterization of the first fungal P450 (CYP5136A3) with a broad catalytic ability to oxidize both alkylphenols (with varying length alkyl side-chain) and PAHs, the two highly toxic environmental contaminants. The information generated is significant considering that the identified P450 is part of the overall enzymatic apparatus in this versatile fungal organism, and thus could be targeted for improved bioremediation applications including those involving mixture of these chemicals. Structure-activity analysis led to the identification of two key catalytic amino acid residues (Trp^129^ and Leu^324^) involved in substrate specificity and/or regio-selectivity for the oxidation activity. This marks the beginning of our understanding of the catalytically significant amino acid residues governing the oxidation of alkylphenols and the tested fused-ring PAHs phenanthrene and pyrene in this class of eukaryotic P450s [16]–[20]. Outcome of the present study opens up avenues for further structure-activity relationship analyses on similar P450s from other fungi as well as higher eukaryotes.

## Supporting Information

Figure S1 Schematic representation of the strategy employed for screening of *P. pastoris* transformants for insertion of *Pc-POR* and *CYP5136A3* (mutated). The primer sequences used are listed in Table 1. Binding site for each primer is shown with an arrow.
(TIF)

Figure S2 LC-ESI/MS analysis profile of the alkylphenols (APs) oxidation products from *P. pastoris PP* WT whole cell assays. Extracted ion chromatograms for APs constructed with cut-off molecular mass of 233.0–233.5 (NP metabolite), 219.0–219.2 (OP metabolite), 205.00–205.13 (HTP metabolite), 177.05–177.10 (PTP metabolite), 163.05–163.10 (BP metabolite), and 149.05–149.10 (PP metabolite). Inset: Extracted ion chromatograms-mass spectrum for the respective AP metabolites. Abbreviations: NP, 4-*n*-nonylphenol; OP, 4-*n*-octylphenol; HTP, 4-*n*-heptylphenol; PTP, 4-*n*-pentylphenol; BP, 4-*n*-butylphenol; PP, 4-*n*-propylphenol.
(TIF)

Figure S3 Structure alignment (Dali server) of the two 3D models of CYP5136A3 built based on human P450s CYP3A4 (PDB ID 1tqn) and CYP1A2 (PDB ID 2hi4) using the Phyre server. Aligned tertiary structures of the models differ by 3.2 Å root mean square deviation (RMSD). Rendered in blue is the model based on the 3A4 template (t3A4); the yellow is based on 1A2 (t1A2).
(TIF)

Figure S4 Structure-based alignment of amino acid sequences and corresponding secondary structure (SS) states of the CYP5136A3 models. SS were computed by the DSSP program and reduced to 3 states: H – α-helices, E – β-sheets, and L – loops. Structurally non-equivalent residues are in lowercase. P450 topology (based on t3A4) is labeled alphabetically for helices and numerically for sheets. Residues labeled with asterisk were mutated in this study (W129A and L324G).
(TIF)

Figure S5 PAH- and APs-oxidizing activities of the wild-type CYP5136A3 (*PP* WT) and mutant form of CYP5136A3 (*PP* W129A). Oxidation of PAHs and APs by *PP* W129A clone was comparable with *PP* WT. The values represent means ± standard deviations for three biological replicates. Abbreviations: Phe, phenanthrene; Pyr, pyrene; NP, 4*-n-*nonylphenol; OP, 4*-n-*Octylphenol; HTP, 4*-n-*heptylphenol; PTP, 4*-n-*pentylphenol; BP, 4*-n-*butylphenol; PP, 4*-n-*propylphenol.
(TIF)
