# Supplementary figures and images for: A Fungal P450 (CYP5136A3) Capable of Oxidizing Polycyclic Aromatic Hydrocarbons and Endocrine Disrupting Alkylphenols: Role of Trp129 and Leu324
Source: PLoS One. 2011 Dec 2;6(12):e28286. doi: 10.1371/journal.pone.0028286 (PMC3229547; doi:10.1371/journal.pone.0028286)

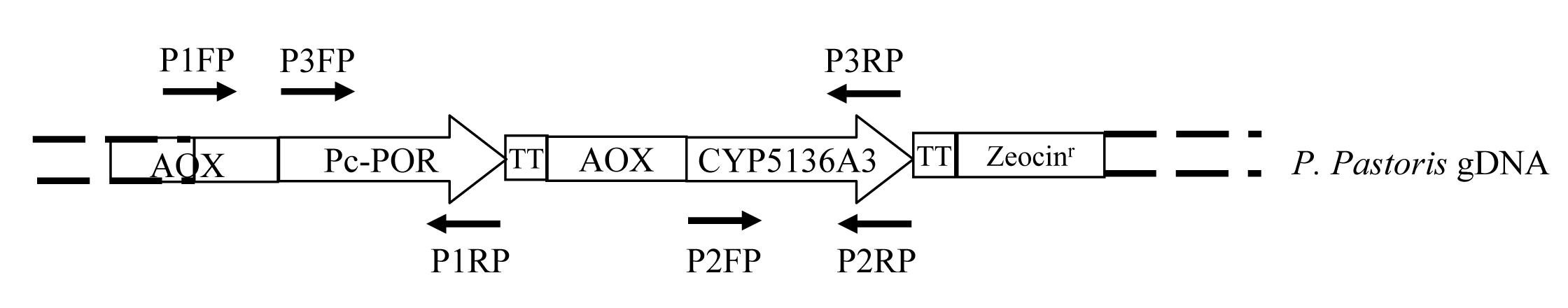

Supplement: Figure S1 — Schematic representation of the strategy employed for screening of P. pastoris transformants for insertion of Pc-POR and CYP5136A3 (mutated). The primer sequences used are listed in Table 1. Binding site for each primer is shown with an arrow. (TIF) [file pone.0028286.s001.tif]

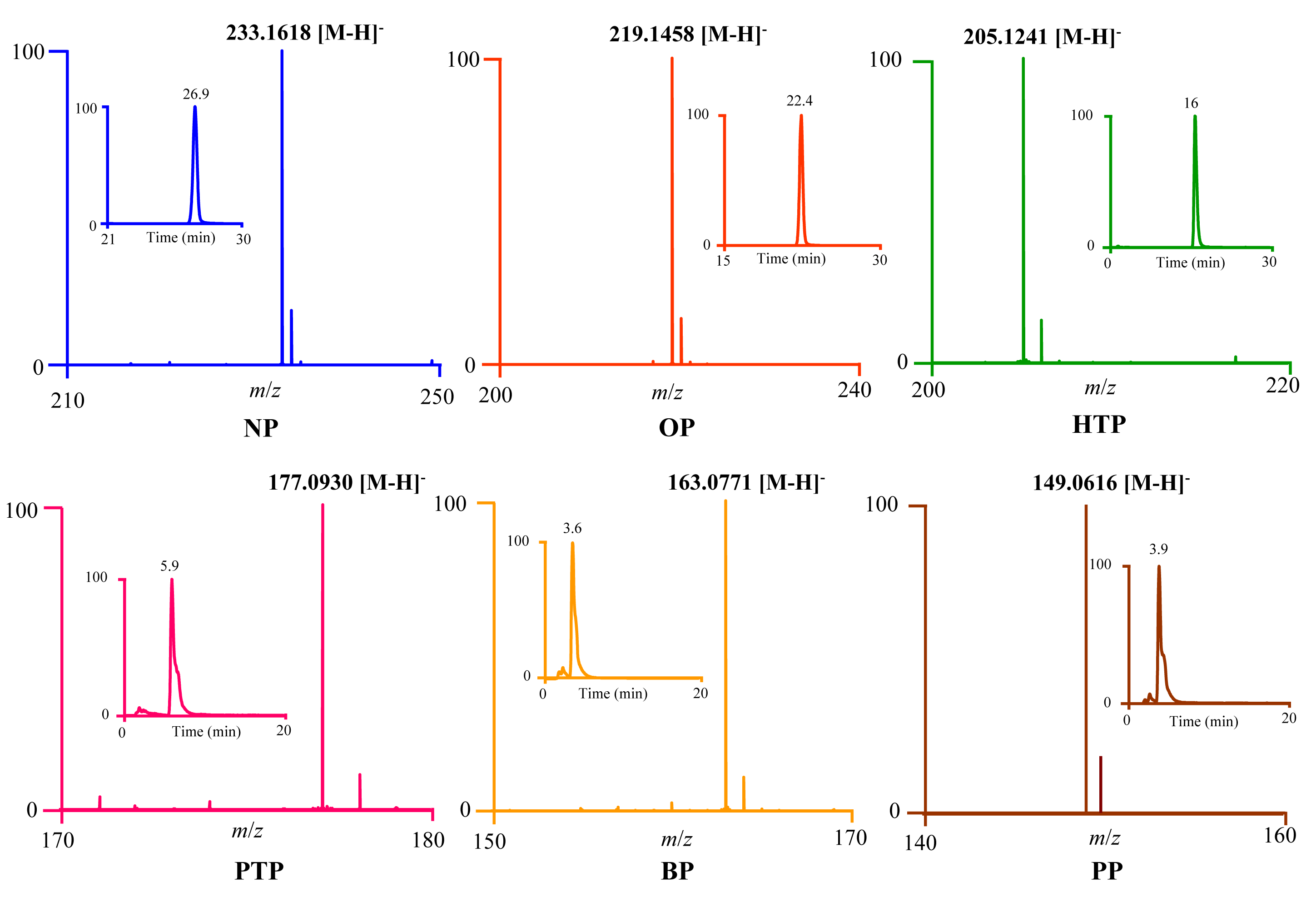

Supplement: Figure S2 — LC-ESI/MS analysis profile of the alkylphenols (APs) oxidation products from P. pastoris PP WT whole cell assays. Extracted ion chromatograms for APs constructed with cut-off molecular mass of 233.0–233.5 (NP metabolite), 219.0–219.2 (OP metabolite), 205.00–205.13 (HTP metabolite), 177.05–177.10 (PTP metabolite), 163.05–163.10 (BP metabolite), and 149.05–149.10 (PP metabolite). Inset: Extracted ion chromatograms-mass spectrum for the respective AP metabolites. Abbreviations: NP, 4-n-nonylphenol; OP, 4-n-octylphenol; HTP, 4-n-heptylphenol; PTP, 4-n-pentylphenol; BP, 4-n-butylphenol; PP, 4-n-propylphenol. (TIF) [file pone.0028286.s002.tif]

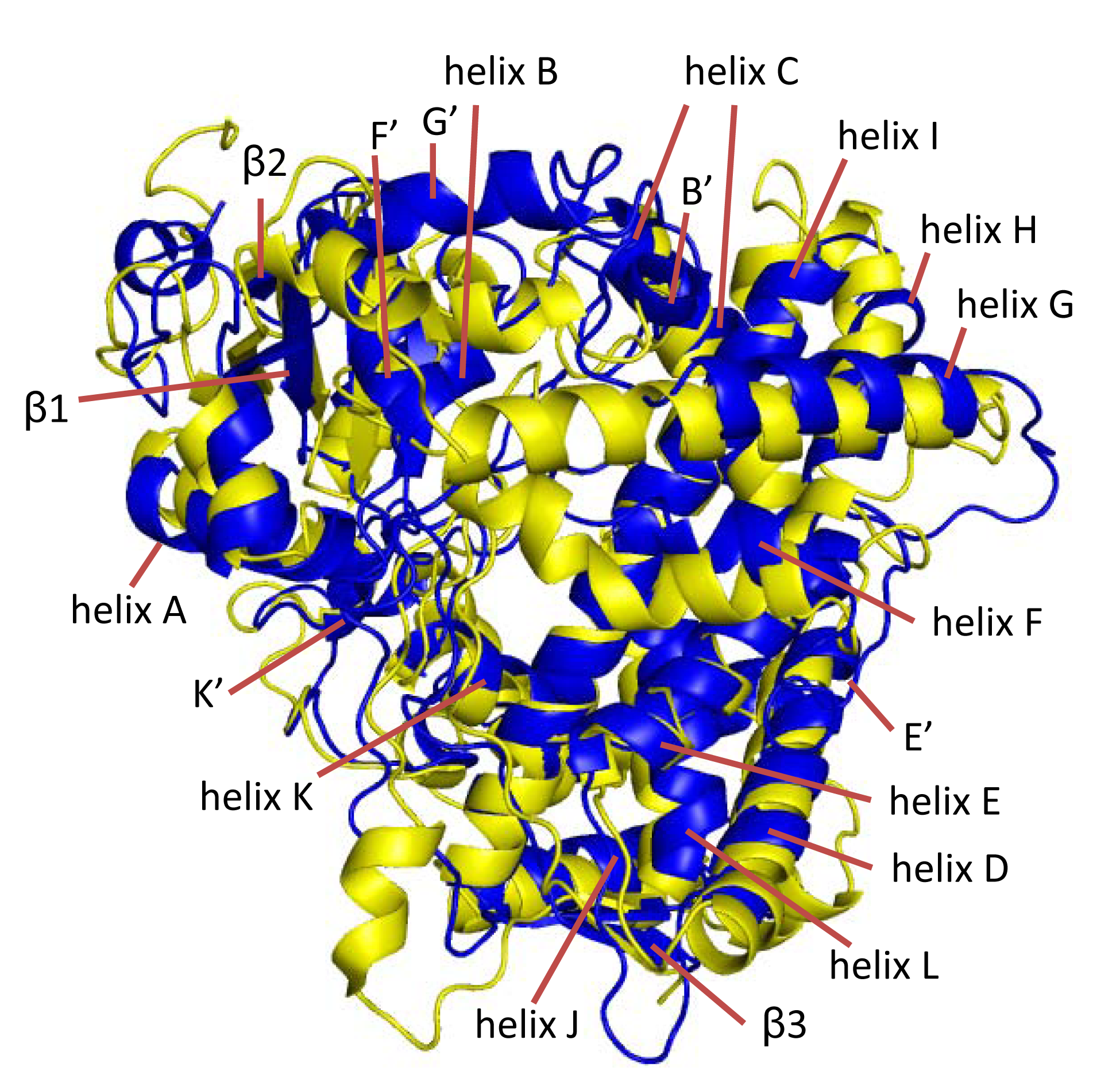

Supplement: Figure S3 — Structure alignment (Dali server) of the two 3D models of CYP5136A3 built based on human P450s CYP3A4 (PDB ID 1tqn) and CYP1A2 (PDB ID 2hi4) using the Phyre server. Aligned tertiary structures of the models differ by 3.2 Å root mean square deviation (RMSD). Rendered in blue is the model based on the 3A4 template (t3A4); the yellow is based on 1A2 (t1A2). (TIF) [file pone.0028286.s003.tif]

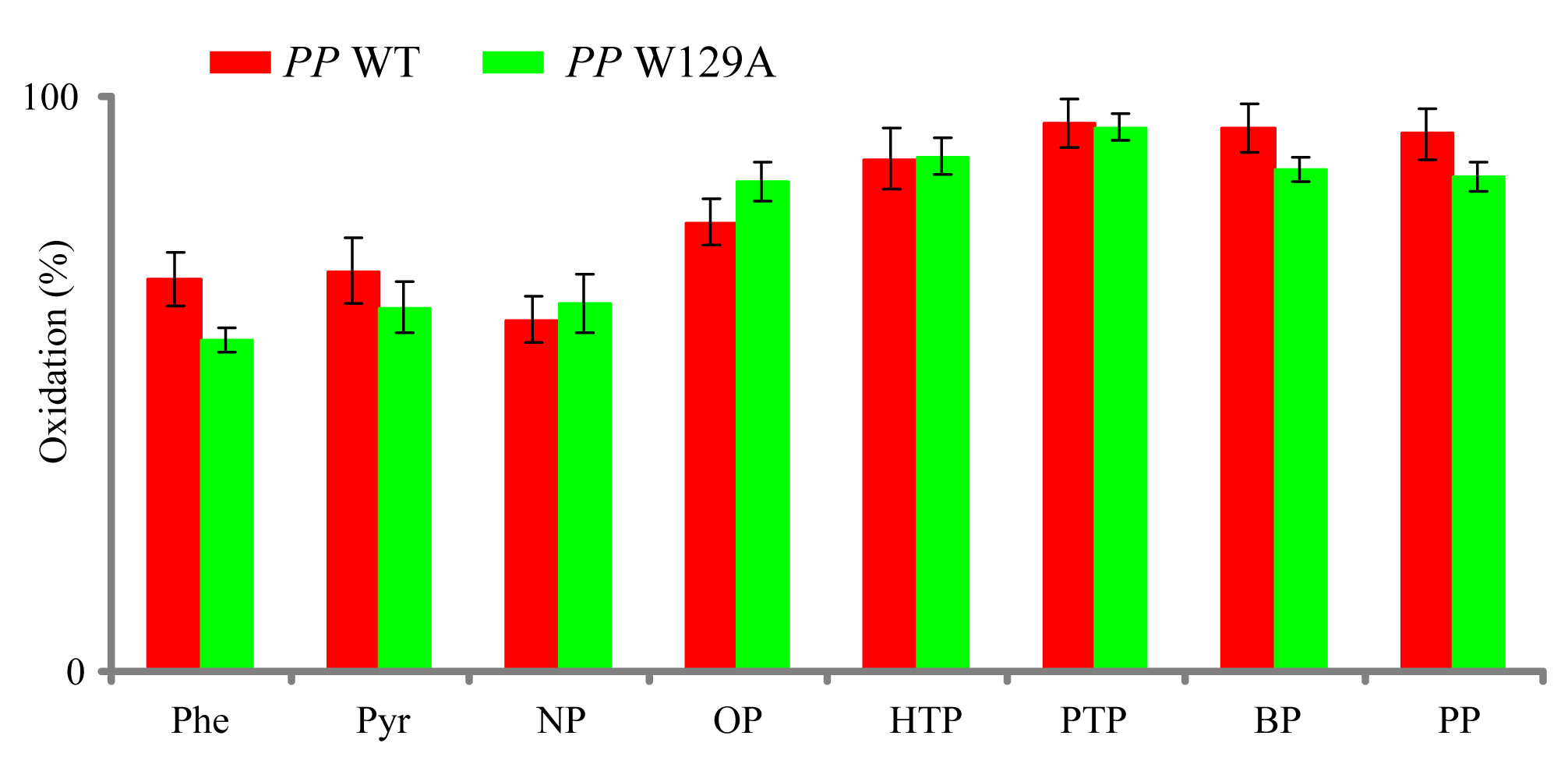

Supplement: Figure S5 — PAH- and APs-oxidizing activities of the wild-type CYP5136A3 (PP WT) and mutant form of CYP5136A3 (PP W129A). Oxidation of PAHs and APs by PP W129A clone was comparable with PP WT. The values represent means ± standard deviations for three biological replicates. Abbreviations: Phe, phenanthrene; Pyr, pyrene; NP, 4-n-nonylphenol; OP, 4-n-Octylphenol; HTP, 4-n-heptylphenol; PTP, 4-n-pentylphenol; BP, 4-n-butylphenol; PP, 4-n-propylphenol. (TIF) [file pone.0028286.s005.tif]
